# Dr. Charles Frere Webb

**Published:** 1911-04-01

**Authors:** 


Dr. Charles Frere Webb, of Basingstoke, Hants, has died at Cley, Norfolk, where he lived after retiring from practice. As M.D. of Durham University, and a Fellow of both Royal Colleges, he had a brilliant record. He studied at King's College and had been M.O.H. at Basingstoke.

